# Purinergic and Calcium Signaling in Macrophage Function and Plasticity

**DOI:** 10.3389/fimmu.2014.00580

**Published:** 2014-11-27

**Authors:** Bimal N. Desai, Norbert Leitinger

**Affiliations:** ^1^Department of Pharmacology, University of Virginia, Charlottesville, VA, USA

**Keywords:** macrophages, calcium, purinergic receptors, inflammation, inflammasome activation

## Abstract

In addition to a fundamental role in cellular bioenergetics, the purine nucleotide adenosine triphosphate (ATP) plays a crucial role in the extracellular space as a signaling molecule. ATP and its metabolites serve as ligands for a family of receptors that are collectively referred to as purinergic receptors. These receptors were first described and characterized in the nervous system but it soon became evident that they are expressed ubiquitously. In the immune system, purinergic signals regulate the migration and activation of immune cells and they may also orchestrate the resolution of inflammation (1, 2). The intracellular signal transduction initiated by purinergic receptors is strongly coupled to Ca^2+^-signaling, and co-ordination of these pathways plays a critical role in innate immunity. In this review, we provide an overview of purinergic and Ca^2+^-signaling in the context of macrophage phenotypic polarization and discuss the implications on macrophage function in physiological and pathological conditions.

## Purinergic Receptors in Macrophages

Purinergic receptors are divided into P1 and P2 receptors. The adenosine receptors are referred to as P1 receptors. P2 receptors are the receptors for adenosine triphosphate (ATP) and can be further subdivided into metabotropic P2Y receptors, which are G-protein-coupled receptors and ionotropic P2X receptors, which are cation-selective ion channels. Macrophages express a wide variety of P2X and P2Y receptors; analysis of mouse macrophages using a variety of techniques indicates the presence of P2X_4_, P2X_7_, P2Y_1_, P2Y_2_, P2Y_4_, and P2Y_6_ receptors (3). Additionally, the expression of P2Y_12_ receptor has been reported in microglia (4) and macrophages (5). Historically, macrophages were also thought to express an additional ATP-sensitive large conductance channel termed P2Z receptor (6). However, this receptor was later shown to be P2X7 by Surprenant et al. (7).

### Release of ATP and other nucleotides into the extracellular space

Cytosolic ATP can be secreted through the release of ATP-loaded vesicles or through the activation of large conductance channels (8). A variety of inflammation-related biological processes result in ATP release from cells, and macrophages respond to this extracellular ATP rapidly. Elegant studies by Ravichandran and colleagues have shown that cells undergoing apoptosis release ATP as a find-me signal that attracts phagocytes (9). In the case of dying cells, the release of ATP and other nucleotides is accomplished through Pannexin 1, a hemi-channel that is activated through caspase-dependent cleavage (10). Interestingly, monocytes stimulated with pathogen-associated ligands or danger molecules, such as uric acid can secrete ATP, which may execute an autocrine signal that results in the activation of inflammasomes and secretion of IL-1β and IL-18 (11). Moreover, the activation of the complement cascade has also been shown to elicit ATP efflux from macrophages and subsequent autocrine activation of the NLRP3 inflammasome (12).

### The function of P2Y receptors in macrophages

In 1989, Dubyak and colleagues showed that treatment of macrophages with extracellular ATP elicits elevations in intracellular Ca^2+^ in a wide variety of myeloid cells but not in lymphocytes. These Ca^2+^-elevations correlated with the hydrolysis of inositol phospholipids suggesting that the ATP receptors were Gq-coupled (13). Over the course of the last two decades, it has become clear that the P2Y receptors on macrophages are Gq and Gi/o-coupled and that they perform a critical function in ATP-responsive chemotaxis. For instance, the chemotaxis of cultured microglia in response to extracellular ATP was shown to be dependent on Gi/o-coupled P2Y receptors by Honda et al. (14). This receptor was later identified as P2Y_12_. Microglia deficient in P2Y_12_ fail to polarize and migrate toward an ATP source *in vitro* and is unable to extend their processes toward sites of brain damage in mice (4). On a related note, microglial phagocytosis is triggered by UDP that is released by damaged neurons and is dependent on P2Y_6_ receptors (15). In monocytes and macrophages, P2Y_2_ plays a crucial chemotactic role in locating apoptotic cells releasing ATP (9). This study used a murine air-pouch model to demonstrate that cell supernatants from apoptotic cells were able to recruit monocytes and macrophages *in vivo* and that this recruitment was diminished in mice lacking the P2Y_2_ receptor. In models of lung inflammation, P2Y_2_ plays a prominent role in the chemotaxis of dendritic cells and eosinophils. Moreover, mice deficient in the P2Y_2_ receptor show reduced airway inflammation in lung inflammation models where ATP has been shown to accumulate in the airways (16). Macrophages navigating in a gradient of C5a secrete ATP and use a purinergic feedback loop that involves P2Y_2_, P2Y_12_, and P1 receptors to migrate (5). However, ATP-triggered chemotactic differences in M1 and M2 macrophages have not been explored and the functional contribution of various P2Y receptors in macrophage phenotypes remains uncharacterized (Table 1).

**Table 1:** **Purinergic receptors and TRP channels in macrophages**.

	Activation	Downstream signaling	Cellular function	Disease model phenotype after targeting
A_1_	Adenosine	Gi-coupled		
A_2A_	Adenosine	Gs-coupled	Augment M2 polarization (63)	Extensive tissue damage and prolonged inflammation (67)
				Agonists induce alleviation of neural inflammation (70)
A_2B_	Adenosine	Gs/Gq-coupled (?)	Augment M2 polarization (63)	Gene deletion exacerbates lung inflammation (71)
				Increased mortality in a sepsis model (72)
A_3_	Adenosine	Gi-coupled	Downregulation of inflammatory cytokines (65, 66)	Reduced arthritis using agonists (67)
P2X_4_	?	?	?	?
P2X_7_	1 mM ATP	Non-selective cation flux	Activation of NLRP3 and caspase-1	Resistance to contact allergen sensitivity (23)
			Lysosomal secretion of cathepsins (27)	Reduced GVHD (24) Resistant to pulmonary inflammation (25)
P2Y_2_	ATP (9)	?	Dendritic cell chemotaxis (9)	Reduced airway inflammation (16)
P2Y_4_	?	?	?	?
P2Y_6_	UDP (15)	Gq-coupled?	Microglial phagocytosis (15)	
P2Y_12_	ATP (4)	Gi/o-coupled (14)	Microglial chemotaxis (4)	
TRPC1	?	Ca^2+^-influx (42)	Unconventional secretion (42)	
TRPV2	?	Ca^2+^-influx (39)	Initiation of phagocytosis (39)	
TRPM2	ROS	Ca^2+^-influx (38)	Chemokine secretion (38)	Reduced neutrophil infiltration and intestinal inflammation (38)
TRPML1	?	Lysosomal Ca^2+^-release (42)	Focal exocytosis during phagocytosis (42)	Decreased bacterial clearance

*?, Unknown*.

### The function of P2X receptors in macrophages

In comparison to P2Y receptors, the P2X receptors have a significantly lower affinity for ATP but their ability to respond to ATP is influenced by the ionic conditions. In the case of P2X_7_, replacement of Na^+^ with K^+^ greatly increases the responsiveness to ATP suggesting a physiological role in damaged tissues with altered ionic conditions (17). Activation of P2X_7_ by high concentrations of ATP mediates caspase-1-dependent cell death accompanied by the release of proinflammatory cytokines, such as IL-1β and IL-18. This process is greatly potentiated in macrophages activated by LPS (18). The processing of IL-1β and IL-18 by caspase-1 is followed by their unconventional secretion with or without accompanying pyroptosis, a caspase-1 mediated pathway of inflammatory cell death. These mechanisms appear to be greatly potentiated by the influx of extracellular Ca^2+^ through the P2X_7_ channels (19). Precisely how Ca^2+^ modulates the machinery mediating the secretion of IL-1β is not clear. Activation of P2X_7_ also induces membrane blebbing and activation of Rho-effector kinases but whether the influx of Ca^2+^ is essential for these processes is also not clear. Concomitantly, P2X_7_ is thought to regulate inflammasome-dependent activation of caspase-1 by mediating K^+^ efflux (20) and through the potentiation of an NFκB-driven transcriptional program (21). In a related process, P2X_7_ has been shown to control the secretion of MHC class II-containing exosomes in NLRP3-dependent but caspase-1-independent manner (22).

Due to the crucial role played by P2X_7_ in the regulation of the NLRP3 inflammasome, it has been implicated as a molecular target in a large variety of inflammatory diseases (Table 1). Mice deficient in P2X_7_ are not sensitized to contact allergens and fail to release IL-1β in response to LPS and ATP (23). This study suggests that the ligation of P2X_7_ by ATP is crucial for initiating skin inflammation. Similarly, P2X_7_ expression on antigen presenting cells appears to have a major impact on graft-versus-host disease (GVHD) (24). How P2X_7_ affects antigen processing and presentation is not clear yet. The P2X_7_-deficient mice have also been shown to be highly resistant to pulmonary inflammation induced by exposure to cigarette smoke (25). In the case of intestinal inflammation, mast cells expressing P2X_7_ have been shown to play a central role in initiating the inflammatory cascade (26). In this case, it seems likely that the influx of Ca^2+^ through P2X_7_ potentiates the degranulation of mast cells. A similar role for P2X_7_-mediated potentiation of lysosomal secretion of cathepsins has been reported in a mouse model of arthritis (27). Recently, it was discovered that monocytes from patients afflicted with Behcets disease, a severe auto-inflammatory disorder, have increased expression of P2X_7_ (28). Recent studies have also implicated P2X_7_ in the modulation of adaptive immunity through the control of antigen presentation on MHC class I molecules (29). In addition to P2X_7_, macrophages also express P2X_1_ channels but the functional significance is not yet clear (30). Although M1 macrophages are more efficient at ATP-induced secretion of IL-1β (31), no significant differences in the expression levels of P2X_7_ have been noted. It has been suggested that P2X_7_ activation is decoupled from IL-1β regulation in M2 macrophages (31).

## Calcium Channels in Macrophages

As non-excitable cells, macrophages rely on Ca^2+^-permeable channels that are not gated by voltage. In addition to P2X channels, macrophages express the store-operated Orai channels and some members of the transient receptor potential (TRP) channel superfamily. The regulation and function of these ion channels remains a mystery and is an emerging topic of significance to inflammation (Table 1).

### Orai channels in macrophages

Historically, the elevations of intracellular Ca^2+^ in macrophages were first observed in response to platelet-activating factor (PAF) (32). In accord with the classic store-operated Ca^2+^-entry, PAF first elicited the mobilization of intracellular Ca^2+^ stores through a Gq-coupled pathway. The emptying of the ER stores was then followed by the opening of the Ca^2+^-permeable channels in the plasma membrane, the so-called CRAC channels (33). For almost two decades, the identity of CRAC channels remained a mystery but we now know their molecular identities as Orai channels (34). Recent discoveries have unraveled the regulatory mechanisms of Orai channels but their functional role in macrophage biology remains undefined. Some observations have linked store-operated Ca^2+^ response to the production of reactive oxygen species (ROS) in macrophages but definitive work and mechanistic insights have not been forthcoming (35). The Ca^2+^-influx necessary for the engulfment of apoptotic cells by macrophages is thought to be mediated by Orai channels and genetic studies in *Caenorhabditis elegans* support this notion (36) but how these channels are activated when macrophages encounter apoptotic cells is not clear and the precise role of Ca^2+^ in the engulfment process has not been clarified. Since P2Y receptors can be Gq-linked, the subsequent depletion of Ca2^+^ stores through IP3 receptors should result in activation of Orai channels. Whether this actually occurs and whether Ca^2+^-influx through Orai channels is critical for the cellular outputs of P2Y receptor stimulation is not yet clear.

### TRP channels in macrophages

The 28 members of TRP channel superfamily are subdivided into TRPC (seven members), TRPV (six members), TRPM (eight members), TRPML (three members), TRPP (three members), and TRPA (one member) families (37). TRP channels are cation-selective channels that are weakly voltage-sensitive and diversely gated by temperature, mechanical force, electrophiles, ligands, and internal cues, such as membrane composition and pH. Recent reports have highlighted the potent functional impact of these channels in macrophages. In monocytes lacking TRPM2, the Ca^2+^-influx in response to ROS is diminished and the cells are unable to produce chemokines necessary for the recruitment of other cells (38). In a mouse model of intestinal inflammation, ulceration and neutrophil infiltration were significantly attenuated in mice lacking TRPM2 (38).

Transient receptor potential channels have also been shown to play a major role in phagocytosis. Macrophages lacking TRPV2 are deficient in the triggering of phagocytosis when they encounter zymosan and IgG opsonized particles (39). Whether this function of TRPV2 is coupled to the influx of Ca^2+^ or other cations is not entirely clear but abnormalities in cytoskeletal rearrangements during phagocytosis were observed and the cells were also found to be deficient in chemotaxis. Mice lacking TRPV2 respond poorly when challenged with *Listeria monocytogenes*. They show increased mortality and greater bacterial load in their organs (39). During phagocytosis, macrophages replenish their membranes through a process termed focal exocytosis. This process was thought to be independent of Ca^2+^ (40), but for the phagocytosis of large particles, the process requires the activity of TRPML1 (41). Xu and colleagues have shown that TRPML1 is a lysosomal channel that is essential for the phagocytosis of large particles. Through the combined use of electrophysiology and live-cell imaging, authors show convincingly that TRPML1 mediates the release of lysosomal Ca^2+^ at the site of membrane uptake during large particle phagocytosis.

Recently, TRPC1 has been shown to play a role in restraining the unconventional secretion of IL-1β (42). Secretion of IL-1β is greatly potentiated after the degradation of TRPC1 by caspase-11 and macrophages lacking TRPC1 show increased secretion of IL-1β in response to inflammatory stimuli. The precise mechanism through which TRPC1 regulates this unconventional secretion machinery is not yet clear.

## Coupling of Purinergic and Calcium Signaling in Macrophages

Extracellular ATP induces Ca^2+^ elevations in myeloid cells through the activation of G_q_-coupled P2Y receptors and Ca^2+^-permeable P2X channels. P2Y receptors have a higher affinity for ATP and can elicit Ca^2+^ mobilization from the intracellular stores at low micromolar concentrations of extracellular ATP (13). In contrast, the P2X channels open at millimolar concentrations of ATP and mediate the influx of extracellular Ca^2+^ and other cations (43). Notably, the mobilization of Ca^2+^ stored in the endoplasmic reticulum is not sufficient for the activation of caspase-1 and secretion of IL-1β. When cells are stimulated with ATP in extracellular medium that is depleted of Ca^2+^, IL-1β secretion is nearly abolished (19). These observations indicate that activation of caspase-1 requires a sustained and more intense rise in intracellular Ca^2+^, which can be mediated by the activation of P2X_7_ channels but not P2Y receptors.

An alternative explanation for differential requirements of Ca^2+^ stores and Ca^2+^-entry in the activation of caspase-1 involves the efflux of K^+^ through the activated P2X_7_ channels. In this model, a concomitant efflux of K^+^ is necessary for the activation of caspase-1 and the rise in intracellular Ca^2+^ without K^+^ efflux is insufficient (44). In any case, although the activation of P2Y receptors by low concentrations of ATP is insufficient to activate caspase-1, the resulting Ca^2+^ oscillations have been shown to promote the transcription of proinflammatory cytokines such as IL-6 (45). The relative contributions of P2X and P2Y receptors in nucleotide-induced Ca^2+^-signaling have not been adequately defined but the use of knockout mice has provided useful insights into this complex aspect of inflammation (46). In myeloid cells, Ca^2+^-dependent activation of PKC plays a pivotal role in the NFκB pathway (47) and the cellular outputs at the site of inflammation are thus likely to be shaped by the purinergic microenvironment. Even in the absence of purinergic signals, Ca^2+^ stores can be mobilized by Toll-like receptors through the activation of tyrosine kinases and phospholipase C (48), but the presence of ATP in the microenvironment likely functions as a potent amplifying mechanism for inflammatory processes.

In addition to the regulation of proinflammatory gene expression and cytokine secretion, Ca^2+^-signaling plays a major role in phagosome maturation. This link is exploited by the internalized mycobacterium for immunoevasive block of phagosome maturation (49). Although the role of Ca^2+^ in phagosome maturation is incompletely defined, it is clear that the lysosomal synaptotagmin VII, a Ca^2+^-sensitive protein, is essential for the fusion of lysosomes with phagosomes (50, 51). Ca^2+^-influx is also essential for the engulfment of apoptotic cells and a subsequent anti-inflammatory response (36). Macrophage phenotypic polarization results in significant differences in the execution of phagocytosis and phagosome maturation but the associated differences in the role of purinergic and Ca^2+^-signals between differentially polarized macrophages remain undefined (52). Ca^2+^-influx has also been shown to be essential to maintain the leading-edge structure in migrating macrophages. In this context, Ca^2+^-influx may be necessary for the activity of PKCα, which is preferentially localized at the leading edge (53).

## Chemotactic and Phagocytic Responses of Macrophages to Extracellular ATP

Macrophages and other cells of myeloid lineage respond at three basic levels to extracellular ATP gradients. First, they migrate toward increasing ATP concentrations; second, they use the ATP gradients emanating from dying cells as a “find-me” signal to locate and phagocytose the cell corpse; and third, at high concentrations of ATP, macrophages respond by robust secretion of proinflammatory cytokines.

Chemotactic responses to ATP were first convincingly demonstrated using cultured microglial cells. Extracellular ATP at micromolar concentrations induced pronounced membrane ruffling, chemokinesis, and chemotaxis (14). This aspect of purinergic response was not confined to ATP gradients emanating from a distant site, indicating ATP acted on cells in an autocrine manner. Indeed, migrating human neutrophils release ATP from their leading edges to amplify and steer their migration using an autocrine feedback loop that involves multiple types of purinergic receptors (54). In macrophages, the chemotactic response to C5a also utilizes an “autocrine purinergic loop” that involves the release of ATP at the leading edge and activation of multiple purinergic receptors (5). In asthmatic airway inflammation, ATP-induced chemotaxis appears to play a critical role in eosinophil and dendritic cell infiltration (55).

The analysis of dendritic cell responses to ATP clearly demonstrates that the chemotactic response to ATP is mechanistically dissociated from other cellular effects of ATP such as secretion of proinflammatory cytokines (16). However, the ATP-induced chemotactic response is intricately connected to the role of purinergic signaling in the location and phagocytosis of apoptotic cells. In the central nervous system, damaged neurons release UDP, which triggers the phagocytic response in neighboring microglial cells (15) and similar mechanisms are likely at work in other tissue-resident macrophages. A definitive role for extracellular ATP as a find-me signal for phagocytes was demonstrated by Elliott et al. (9). The release of ATP and UTP is dependent on the activation of caspases and Pannexin 1 during the early stages of apoptosis and the concentration gradient generated by such release is highly efficient at recruiting monocytes and macrophages. Whether different macrophage phenotypes migrate differently in response to ATP gradients has not been explored. Phagocytosis of dying cells that release ATP serves an anti-inflammatory role, and phagocytotic capacity is drastically inhibited in Mox macrophages that accumulate at the sites of oxidative tissue damage (56). In this context, it would be interesting to know whether alternatively activated M2 or M2-like macrophages show any significant specialization in locating dying cells using the ATP gradients.

## Extracellular ATP as a Trigger for Proinflammatory Cytokine Secretion and Pyroptosis

Sustained exposure to relatively high concentrations of ATP has been shown to be a critical signal for the secretion of proinflammatory cytokines, such as IL-1β and IL-18. The unconventional secretion of these cytokines is accomplished through proteolytic processing by caspase-1. The activation of caspase-1 is regulated by large multimeric complexes called inflammasomes and the activation of one such inflammasome, the NLRP3 inflammasome, is highly sensitive to the presence of extracellular ATP. Early evidence for ATP-induced maturation of IL-1β came from studies involving apoptosis of peritoneal exudate cells when exposed to high concentrations of extracellular ATP. This form of apoptosis, which we now refer to as pyroptosis, was accompanied by proteolytic processing and release of IL-1β (57), and was especially pronounced in LPS-stimulated mouse peritoneal macrophages where exposure to millimolar concentrations of ATP resulted in rapid processing and release of IL-1β. It was further demonstrated that exposure to high concentrations of extracellular K^+^ prevented the processing and release of IL-1β (58), suggesting that depletion of intracellular K^+^ was essential for ATP-induced IL-1β processing. Subsequent studies of this phenomenon were greatly facilitated by the isolation of the human monocytic cell line THP-1, which was shown to be highly sensitive to purinergic stimulation of IL-1β processing. At least in human monocytes, the purinergic activation of IL-1β processing and secretion is also accompanied by release of proteolytically activated caspase-1 and a commitment to cell death (59).

A study conducted by Dixit and colleagues tested the role of channel-mediated ATP release by characterizing Pannexin 1-deficient mice in the context of inflammasome activation (60). Authors show that the activation of caspase-1 and secretion of IL-1β in response to a wide variety of stimuli including ATP is normal in Pannexin-1-deficient macrophages. In contrast, Pannexin 1-deficient thymocytes failed to recruit macrophages after undergoing apoptosis. Overall, these studies indicate that ATP released through Pannexin 1 is sufficient to reach extracellular concentrations that are of functional relevance to chemotaxis. However, the activation of caspase-1 typically requires high concentrations of ATP that are unlikely to be reached when cytosolic ATP is released through Pannexin 1. The role of vesicular release of ATP in the autocrine activation of inflammasome has not yet been ruled out and that may hold the key to reconcile these studies. A key component of vesicular ATP release is the vesicular nucleotide transporter VNUT (also known as SLC17A9), which is responsible for the accumulation of ATP into secretory vesicles (61). The human monocytic cell line THP-1 has been shown to express VNUT, which mediates the rapid secretion of ATP in response to LPS treatment (62). The function of vesicular ATP secretion in mouse macrophages and its physiological significance have not been reported yet. It is also not clear whether M1 and M2 macrophages exhibit mechanistic and functional differences in ATP release mechanisms.

The mechanisms through which activated caspase-1 and IL-1β are secreted have remained unclear but there is evidence for the involvement of Ca^2+^-influx elicited by ATP (19). Interestingly, although high concentrations of ATP are required for caspase-1 activation, low concentrations of ATP (10 μM) or UTP (10 μM) are sufficient to induce oscillations in intracellular Ca^2+^ and increased transcription of the proinflammatory cytokine IL-6 (45). An autocrine role for ATP-induced activation of inflammasome has also been suggested in the case of primary human monocytes that are stimulated by danger-associated uric acid. According to this model, the activation of inflammasome is dependent upon the initial release of ATP, which then acts on purinergic receptors in an autocrine manner. Whether such secretion results in local ATP concentrations that are high enough to activate the inflammasomes is not clear (11). In addition to the regulation of IL-1β and IL-18 secretion, ATP has also been shown to regulate the secretion of lysosomal cathepsins (27). These proteases are involved in the degradation of extracellular matrix and can result in auto-inflammatory tissue damage. Recently, ATP was shown to potentiate the release of IFNβ in LPS-stimulated macrophages (30).

With respect to macrophage phenotypes, a recent study provided evidence that inflammatory M1 macrophages are more sensitive to ATP and more efficient at ATP-induced IL-1β release when compared to M2 macrophages (31). While the physiological significance of these findings remains to be elucidated, this study provides the initial exploratory foray into these outstanding questions pertaining to how macrophage polarization fine-tunes the sensitivity to the purinergic microenvironment.

## The ATP Metabolite Adenosine Regulates the Resolution of Inflammation

Many cells express membrane-bound ectonucleotidases that convert the extracellular ATP and ADP to adenosine. A common pathway involves the conversion of ATP and ADP to AMP by CD39 and subsequent conversion of AMP to adenosine by CD73. These enzymatic biochemical conversions have potent implications for the termination of inflammatory response due to the reduction in ATP levels. More significantly, adenosine serves as a ligand for G-protein coupled adenosine receptors or P1 receptors on myeloid cells (Table 1). The four P1 adenosine receptors (A_1_, A_2A_, A_2B_, and A_3_) transmit a “calm down” signal that may orchestrate the resolution of inflammation, a process conceptually different from anti-inflammatory signals that restrain the initiation of inflammatory process by preventing the recruitment and activation of immunocytes. Pertinently, extracellular adenosine has been shown to augment the polarization of macrophages toward the M2 phenotype (63).

Early studies showed that adenosine inhibited the secretion of TNFα, IL-6, and IL-8 by LPS-activated human monocytes (64). Subsequently, it was shown that the adenosine receptor A_3_ plays a major role in downregulating the synthesis of proinflammatory cytokines in monocytes and macrophages in response to extracellular adenosine (65, 66). Synthetic agonists of A_3_ adenosine receptor have been shown to have potent therapeutic effects in various mouse models of rheumatoid arthritis (67). A similar role for the A_2A_ adenosine receptor has also been reported. Mice deficient in the A_2A_ receptor show extensive tissue damage and prolonged inflammation to sub-threshold doses of inflammatory stimuli in three different disease models (68). In the context of infectious diseases and polymicrobial sepsis, A_2A_ receptors are required for the control of IL-10 production by alternatively activated macrophages (69). The A_2A_ receptors also play an anti-inflammatory role in neuroinflammation. The ligation of A_2A_ receptors on the activated microglia has been shown to retract their processes and initiate the resolution of inflammation in the brain (70).

In the case of endotoxin-induced lung injury, the pharmacological inhibition or genetic deletion of A_2B_ receptors greatly exacerbates lung injury. In contrast, the A_2B_ receptor agonist attenuates endotoxin-induced lung inflammation (71). The role of A_2B_ receptors in dampening endotoxin-induced inflammation is evident in mouse models of polymicrobial sepsis. Deletion of A_2B_ receptors greatly increases the mortality of mice from cecal ligation and puncture-induced sepsis (72). The A_2B_-deficient mice showed increased levels of proinflammatory cytokines and chemokines in the serum, coincident with augmented activation of NFκB and p38 in the spleen.

In summary, the adenosine receptors provide crucial information to monocytes and macrophages and calibrate their response to the complex mix of purinergic stimuli in the inflammatory microenvironment (73). In this context, the ratio of ATP and adenosine may provide the crucial cues necessary for the polarization of macrophages toward the M2 phenotype induced by IL-4. The role of A_2A_ and A_2B_ receptors to augment this process of M2 polarization has been illuminated (63) but surprisingly, a recent study shows that adenosine may control this process independent of IL-4 (74). Similarly, a recent study provides evidence suggesting a role for adenosine in IL-10-induced STAT3 activation in alternatively activated phenotype termed M2c (75). Manipulation of ATP and adenosine levels in the tissue microenvironment is thus likely to emerge as a potent mechanism to guide the plasticity of macrophages and holds clinical potential for therapeutic intervention. This concept may find traction in a large variety of diseases where inflammation plays a major pathological role. A recent preclinical study exemplifies the application of this strategy in the treatment of osteolysis; the authors show that activation of A_2A_ receptors prevents wear-induced osteolysis (76).

## Extracellular ATP Plays a Prominent Role in Inflammatory Diseases

Some of the earliest evidence indicating a role for extracellular ATP in monocyte and macrophage function came from the findings that the cells of the myeloid lineage exhibit rapid elevations in intracellular Ca^2+^ when treated with micromolar amounts of ATP (13). The *in vivo* significance of this finding was further highlighted by the studies of Bertics and colleagues who showed that LPS-induced activation of macrophages was greatly enhanced by extracellular ATP (77) and that mice treated with the adenine nucleotide analog 2-methylthio-ATP were protected from endotoxic shock (78). Subsequent studies indicated a potent role for purinergic signals in controlling the inflammatory gene expression in response to LPS stimulation (79). The extensive contribution of purinergic signaling in inflammatory processes has now been established in a wide variety of pathologies (2), some of which are outlined below.

The tissue-resident macrophages of the central nervous system, the microglia, have been shown to be especially sensitive to extracellular ATP. *In vitro* studies with cultured microglia revealed that extracellular ATP and ADP stimulate chemotaxis and morphological changes (14). Local trauma in the brain, which results in cell death, is thought to increase the extracellular levels of ATP significantly and elegant studies using multiphoton imaging have demonstrated that microglia respond rapidly to local injury through dynamic changes in their morphology. The convergence of microglial processes at the injury site could also be stimulated by local injection of ATP and this response was demonstrated to be highly sensitive to the presence of ATP-hydrolyzing enzymes and blockers of purinergic receptors (80).

In the lungs of asthmatic patients, allergic challenges cause rapid accumulation of ATP and this has been modeled successfully in mice using experimentally induced asthma (55). Interestingly, hydrolysis of ATP in the airways through the application of apyrase greatly reduces eosinophil infiltration, production of Th2 cytokines, and bronchial hyper-reactivity. Corollary experiments show that exogenous ATP potentiates airway inflammation (55) and similar findings have demonstrated a role for purinergic signals in cigarette smoke-induced inflammation and emphysema (25). The significance of purinergic signals in allergic reactions is also evident in the skin. The accumulation of ATP in response to contact allergans is critical for the production of inflammatory cytokines by the myeloid cells and the subsequent sensitization process (23). Accumulation of ATP has also been observed in the ascites of patients and mice undergoing GVHD. In mice, the severity of GVHD is greatly reduced by neutralizing the ATP or by blocking purinergic signaling (24). Furthermore, recent studies have also implicated extracellular ATP in the development of intestinal inflammation in patients with Crohn’s disease. In corresponding mouse models, blocking purinergic signaling greatly reduces the activation of intestinal mast cells and thereby blocks the subsequent rise in proinflammatory cytokines and leukotrienes (26).

In summary, studies in human beings and mice provide conclusive evidence that purinergic signals play a major role in inflammation and tissue injury. Purinergic receptors are expressed ubiquitously and a comprehensive understanding of how purinergic signals influence the physiology and pathology is still in rudimentary stages. Purinergic control of macrophage function promises to play a central role in these processes and understanding the effects of purinergic signals on macrophage function provides an immediate window toward therapeutic intervention.

## Concluding Remarks

Our understanding of the fundamental role played by purinergic and Ca^2+^ signaling in macrophage activity is increasing rapidly but the signaling mechanisms that drive specific cellular outputs still remain largely enigmatic. The close coupling of purinergic stimulation and Ca^2+^ influx suggests that the purinergic receptors, Orai channels, and TRP channels function in a co-ordinated network that responds rapidly to the changes in the inflammatory microenvironment. By virtue of being excellent drug targets, purinergic G-protein coupled receptors and ion channels offer an enticing pharmacological path to shape the plasticity of macrophage function in various diseases. To make this a reality, we will need to develop experimental models where the influence of the purinergic microenvironment and the resulting Ca^2+^-dynamics in macrophages can be interrogated *in situ*. All indications are that we have only just scratched the surface in this exciting area of innate immunity.

## Conflict of Interest Statement

The authors declare that the research was conducted in the absence of any commercial or financial relationships that could be construed as a potential conflict of interest.
